# Prescription Opioid Usage and Abuse Relationships: An Evaluation of State Prescription Drug Monitoring Program Efficacy

**DOI:** 10.4137/sart.s2345

**Published:** 2009-05-01

**Authors:** Richard M. Reisman, Pareen J. Shenoy, Adam J. Atherly, Christopher R. Flowers

**Affiliations:** 1 Gwinnett Hospital System Pain Management Center, Lawrenceville, GA, U.S.A.; 2 School of Medicine, Emory University, Atlanta, GA, U.S.A.; 3 Department of Health Policy and Management, Rollins School of Public Health, Emory University, Atlanta, GA, U.S.A.

**Keywords:** prescription opioids, substance abuse, PDMP

## Abstract

**Context:**

The dramatic rise in the use of prescription opioids to treat non-cancer pain has been paralleled by increasing prescription opioid abuse. However, detailed analyses of these trends and programs to address them are lacking.

**Objective:**

To study the association between state shipments of prescription opioids for medical use and prescription opioid abuse admissions and to assess the effects of state prescription drug monitoring programs (PDMPs) on prescription opioid abuse admissions.

**Design and Setting:**

A retrospective ecological cohort study comparing state prescription opioid shipments (source: Automation of Reports and Consolidated Orders Systems database) and inpatient admissions for prescription opioid abuse (source: Treatment Episode Data Set) in 14 states with PDMPs (intervention group) and 36 states without PDMPs (control group) for the period 1997–2003.

**Results:**

From 1997 to 2003, oxycodone, morphine, and hydrocodone shipments increased by 479%, 100%, and 148% respectively. Increasing prescription oxycodone shipments were significantly associated with increasing prescription opioid admission rates (p < 0.001). PDMP states had significantly lower oxycodone shipments than the control group. PDMP states had less increase in prescription opioid admissions per year (p = 0.063). A patient admitted to an inpatient drug abuse rehabilitation program in a PDMP state was less likely to be admitted for prescription opioid drug abuse (Odds ratio = 0.775, 95% Confidence Interval 0.764–0.785).

**Conclusions:**

PDMPs appear to decrease the quantity of oxycodone shipments and the prescription opioid admission rate for states with these programs. Overall, opioid shipments rose significantly in PDMP states during the study period indicating a negligible “chilling effect” on physician prescribing.

## Introduction

It is estimated that seventy-five million Americans suffer from chronic pain every day. Until the mid 1990’s, prescription opioids were accepted only as a treatment for cancer pain. However, by the late 1990’s, legitimate medical usage of prescription opioids for the treatment of acute, cancer, and non-cancer pain became a basic standard of care and a national public health goal in the United States.

Unfortunately, there also has been a dramatic increase in the incidence of prescription opioid abuse.^1^,^2^ The possible sources of abused prescription opioids are physician prescriptions, theft (from retail pharmacies, manufacturing plants, and distribution centers), pharmacy websites selling prescription opioids without legitimate physician prescriptions, and illegal transport of prescription opioids into the United States. The Controlled Substances Act passed by the Congress of United States identifies and regulates the controlled substances included in each of five schedules. These schedules have differences in the criteria for a substance to be controlled/excluded from them.^3^ The commonly held, but unproven belief, that a significant proportion of physician’s opioid prescriptions are being diverted and abused has resulted in thirty eight states creating statutes establishing prescription drug monitoring programs (PDMPs) with twenty-nine being operational. Each state’s program maintains a central database of a specific combination of controlled substance prescriptions schedules I, II, III, IV, and/or V written by physicians.^4^ The intent of this database is to more easily identify “doctor shoppers,” patients who fraudulently obtain potentially addictive opioid prescriptions from unsuspecting physicians to either abuse the prescription opioids themselves or to sell the opioids. Access to the PDMP database allows physicians, pharmacists, and law enforcement officials to identify doctor shoppers by documenting their unusual prescription patterns thus, preventing them from obtaining prescription opioids for non medical usage.

Although PDMPs were created with the premise that increasing medical usage of prescription opioids contributes to increasing prescription opioid abuse, there are no studies in the literature demonstrating a statistically significant association between the two. Also, there have been very few statistical assessments of the impact of PDMPs on legitimate opioid prescribing (medical usage) or on prescription opioid diversion and abuse.^5^ Although the United States General Accounting Office reported to Congress that PDMPs are successful, 6 there are concerns that PDMPs have a “chilling effect” (deterring physicians from prescribing opioids to successfully treat a patient’s pain) due to the potentially negative influence of drug enforcement agents monitoring their prescribing behaviors.^8^ The purpose of this study is to examine the association between shipments of prescription opioids for medical usage and prescription opioid abuse admissions, and to assess the impact of PDMPs on prescription opioid usage and prescription opioid abuse.

## Methods

### Conceptual model

Medical usage of prescription opioids is represented by the yearly per capita state shipments of prescription opioids in the U.S. as documented by the Automation of Reports and Consolidated Orders Systems (ARCOS), a federal surveillance system of all controlled substance shipments maintained by the Drug Enforcement Administration (DEA) Office of Diversion and publicly available.^9^ Prescription opioid abuse is represented by the yearly state inpatient admission rates for prescription opioids as documented in the Treatment Episode Data Sets (TEDS), a federal surveillance system of all drug admissions into publicly funded drug rehabilitation facilities maintained by the Drug and Alcohol Services Information System and publicly available.^10^

**Figure 1:**
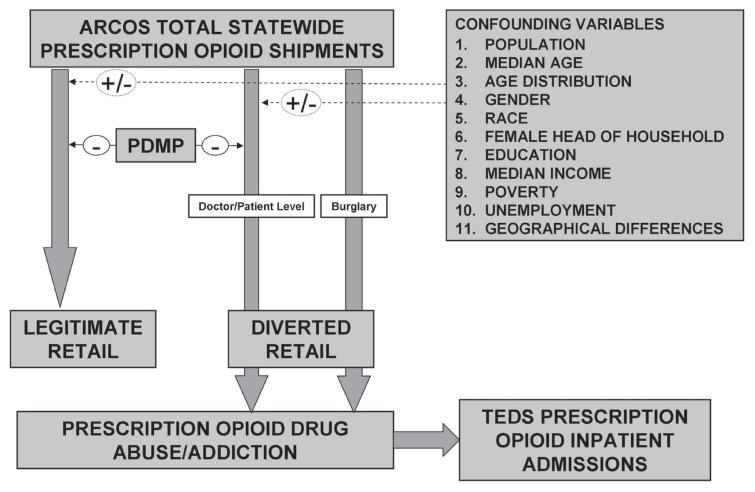
The relationships between prescription opioid state shipments and legitimate use/diversion/abuse and the impact of confounding factors. This is a logic model we used to describe the hypothesized associations.

Figure 1 displays the hypothesized relationships between prescription opioid shipments, inpatient admissions for prescription opioid abuse, PDMPs, and socioeconomic variables that have been shown to be potentially confounding factors.^11^ We also included two potentially confounding variables to account for geographic differences in PDMP and non-PDMP groups. Increases in prescription opioid shipments can lead to increased diverted prescriptions leading in turn to increased prescription opioid inpatient admissions. PDMPs can have a negative effect on diverted and abused prescription opioids which can lead to both decreased prescription opioid shipments secondary to curtailed demand by doctor shoppers and to decreased prescription opioid admissions secondary to a decrease in fraudulently obtained opioid prescriptions. PDMPs also may have a negative or chilling effect on the magnitude of legitimate physician prescribing. This negative effect would lead to a decrease in the ARCOS oxycodone, hydrocodone, morphine, and codeine shipments into states since fewer physicians would be prescribing them.

Finally, burglary anywhere along the chain could affect both state shipments and abuse admissions. It is not possible to distinguish in the model between diversion of opioids secondary to burglary from pharmacies and diversion of opioids secondary to misused physician prescriptions. We are making the assumption that there will be some uniformity between these two types of diversions in PDMP and non-PDMP states. This assumption may not be accurate. Burglary has been described as a real source of diversion.^2^

Using the ARCOS prescription opioid state shipment data, and the TEDS prescription opioid inpatient admissions data, we address three research questions in this paper: 1) Are increasing prescription opioid shipments to states associated with increasing prescription opioid abuse admissions? 2) Do PDMPs reduce prescription opioid abuse admissions? 3) Do PDMPs exert a chilling effect on physician prescribing practices thereby reducing shipments of prescription opioids for medical use?

### Data sources and collection

We used a classic ecological analytic design with each state being an ecological unit, and analyzed the association between medical usage of prescription opioids and prescription opioid abuse. PDMPs maintain databases that include the prescription date, the prescribing health professional, the patient’s name and address, and the medication’s name, dosage, amount prescribed, and the dosing information. PDMPs have been enacted in 38 states and were operational in 32 states as of November 2008.^12^ We focused on the time period from 1997 to 2003 because it had a stable group of PDMP states. Fourteen states had active programs during this period with nine reporting schedule II only (CA, HI, IL, IND, MA, MI, NY, OK, and TX) and five reporting schedule II and III (ID, KY, NV, RI, and UT).^4^,^7^ MEDCO, the largest retail mail order pharmacy in the U.S. resides in Nevada, a PDMP state, thereby skewing the state’s shipment of all four opioids upwards. Contact with the Freedom of Information Action division^13^ revealed that segregating mail order retail pharmacy shipments from the ARCOS datasets was not possible. Therefore, when performing the analytic statistical comparisons between the two groups with respect to their prescription opioid shipments we excluded Nevada from the PDMP group (PDMP 13).

The United States DEA Department of Diversion maintains the ARCOS database which provides statistics on statewide shipments of prescription opioids yearly.^14^,^15^ In our study, medical usage was quantified by using each state’s prescription opioid shipments in grams of opioid per 100,000 population per year from 1997 to 2003 as detailed in the ARCOS database.^15^

In this study, we analyzed the state shipments of oxycodone and morphine (Schedule II) and hydrocodone and codeine (Schedule III) opioids. The practical difference in these schedules is that refills can be written or called into pharmacies for schedule III medications whereas schedule II medications can not have refills or be called into pharmacies. These schedule differences were made for three reasons. First, it was initially thought that codeine and hydrocodone were “weak” opioids and had a lower potency then oxycodone and morphine. Subsequently, it has been realized that hydrocodone is close to being equipotent to oxycodone and has a high abuse potential. Codeine is still considered to be a “weak” opioid. Second, hydrocodone and codeine are always combined with acetaminophen when they are in tablet form in the United States. This hypothetically limits the maximum dosage a person can use of these two medications since acetaminophen has a maximum allowable dosage of 4 grams. In reality, a person addicted to hydrocodone will ignore this maximum allowable dosage and will ingest whatever dosage of the opioids necessary to generate the “high” they are seeking. Third, both oxycodone and morphine are available in much higher tablet dosages than codeine and hydrocodone.

The TEDS database is maintained by the Drug and Alcohol Services Information System which is part of the Substance Abuse and Mental Health Services Administration, an agency of the U.S. Department of Health and Human Services.^16^,^10^ It is created from reports on every patient admitted to publicly funded drug rehabilitation units in every state.^10^ Each patient’s record contains the gender, age, state of residence and details of the drugs of abuse on admission. Prescription opioid abuse was quantified by using the number of inpatient prescription opioid rehabilitation admissions per 100 total drug rehab admissions and the percent change in the state prescription opioid rehabilitation admission rates since 1997.

To study the potential chilling effect of PDMPs on legitimate prescription opioid usage and their effect on opioid admissions, we compared these outcomes for the states with PDMPs (intervention group) and the states without PDMPs (control group). To further assess the success of PDMPs in decreasing prescription opioid abuse we conducted an analytical cross sectional study in 2003 assessing the incidence of inpatient prescription opioid abuse admissions in patients in PDMPs versus patients in non-PDMP states.

### Statistical analysis

We hypothesized that 1) increasing medical usage of prescription opioids is associated with increasing prescription opioid abuse, 2) PDMPs do not exert a chilling effect on prescription opioid shipments, and 3) PDMPs decrease prescription opioid abuse.

Scatter plots were created and linear regressions were calculated to assess the association between these variables. A time series line graph compared the PDMP and control groups for state shipments of oxycodone, hydrocodone, morphine and codeine, the prescription opioid rehabilitation admission rates and admission rate changes since 1997 for the 1997 to 2003 time period. A time series linear regression analysis was performed to determine if differences between the two groups were statistically significant. When comparing the prescription opioid admission rate changes, the PDMP 14 group was used because Nevada’s mail order retail shipments did not affect its’ prescription opioid admission rate statistics. Robust standard error was used to correct for clustering of observations within the states during the 7 year study period.

Using variables identified by Kallan,^11^ we included the year 2000 census statistics for nine socioeconomic variables in our multivariate linear regression analysis to control for potential confounders: total U.S. population, median age, race, gender, educational level, median income, poverty level, female head household, and unemployment rate for each state. Also included in the multivariate linear regression as potential confounders were the geographical variables population density and housing density for each state. PDMP states differed from non-PDMP state in the median age of the population, percentage of Hispanic/Latino individuals, population density, and housing density (Table 1). The analytic cross sectional study provides the odds that a patient admitted to an inpatient drug rehabilitation program in a PDMP state versus a non-PDMP state would be abusing prescription opioids.

## Results

**Figure 2:**
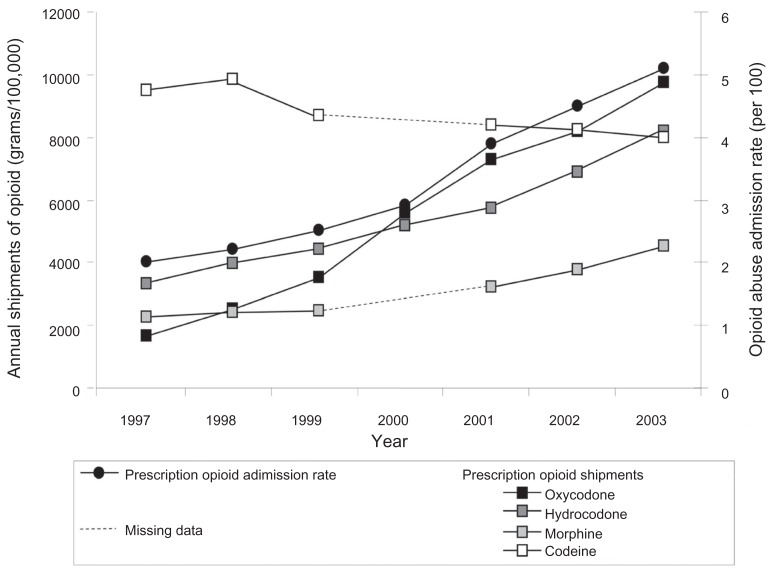
National yearly oxycodone, morphine, hydrocodone, and codeine shipments and prescription opioid admission rates. **Note:** The annual shipments of codeine and morphine were not reported for the year 2000 in the Automation of Reports and Consolidated Orders Systems database.

During the period from 1997 to 2003, there were marked increases in the national yearly shipments of oxycodone (479%, from 1680 to 9728 grams/100,000 population), hydrocodone (148%, from 3291 to 8173 grams/100,000 population), and morphine (100%, from 2249 to 4496 grams/100,000 population), while codeine shipments decreased by 16% (from 9512 to 7963 grams/100,000 population; (Fig. 2). During the same period, the prescription opioid inpatient admission rate more than doubled from 2 to 5.1 opioid rehabilitation admissions/100 total drug rehabilitation admissions (Fig. 2).

**Figure 3:**
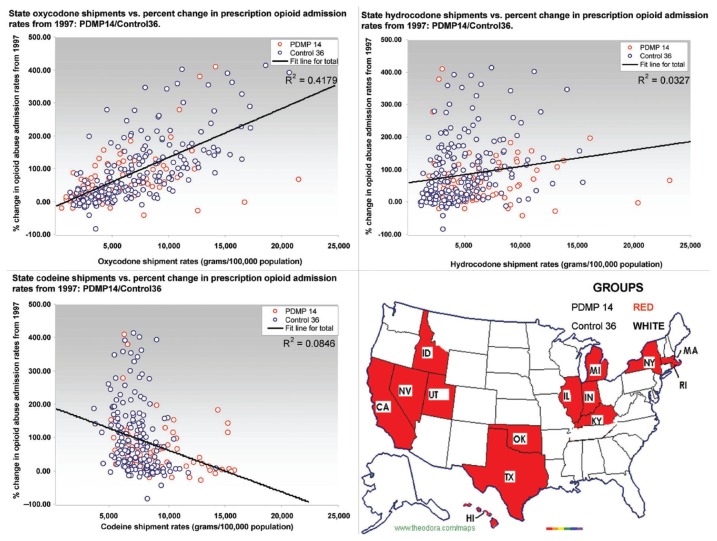
State shipments of opioids and the percent change in opioid admission rates from 1997. Scatterplots of the state shipments of oxycodone, hydrocodone and codeine against the percent increase in opioid abuse admissions from 1997. Each scatterplot also shows a fitted line of regression for all 50 states combined. Also shown is a map of United States depicting the PDMP and control states.

Comparing state prescription opioid shipments to the prescription opioid admission rate change since 1997 (Fig. 3) revealed that the increase in oxycodone shipments had the strongest positive association with the increase in the prescription opioid abuse admission rate (R^2^ = 0.418). Increases in shipments of hydrocodone and morphine were weakly associated with increases in the prescription opioid admission rate (R^2^ = 0.032 and 0.191 respectively). Codeine shipments showed a weak negative association with the admission rate changes (R^2^ = 0.085). The increase in oxycodone shipments contributed to 41.8% of the increase in the prescription opioid admission rate since 1997.

**Figure 4:**
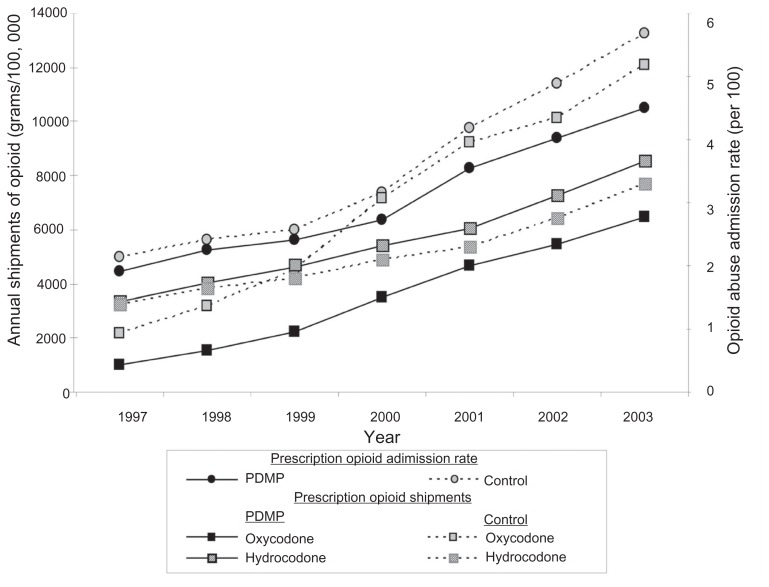
Yearly oxycodone and hydrocodone shipments and inpatient prescription opioid admissions rates in PDMP and control states (1997–2003). **Note:** Nevada was not included in the PDMP group.

Figure 4 compares the group of 13 PDMP states with the control group of 36 states for mean oxycodone and hydrocodone shipments, and the opioid abuse admission rates. The PDMP group demonstrated a 553% increase in oxycodone shipments and 158% increase in hydrocodone shipments from 1997 to 2003. The control group demonstrated a 456% increase in oxycodone shipments and a 138% increase in the hydrocodone shipments. 2003 oxycodone shipments were lower in the PDMP group compared to the control group (6463 vs. 12088 gms/100,000 population), however, hydrocodone shipments were higher in the PDMP group as compared to the control group (8542 vs. 7711 gms/100,000 population). Time series linear regression demonstrated a significant reduction in the rise of oxycodone shipments for the PDMP group compared with control (beta = −370.9, p = 0.019). Not only did PDMP states have lower increases in opioid admissions during this period compared to the control group, but the gap widened with each successive year.

A multivariate linear regression was initially performed using the percent change in opioid admissions since 1997 as the dependent variable and the four opioids as the independent variables (Table 2). Only oxycodone and codeine demonstrated statistical significance. We then performed a multivariate linear regression using only oxycodone and codeine along with the eleven potentially confounding factors as independent variables and the percent change in opioid admissions since 1997 as the dependent variable (Table 3). None of the eleven confounding factors predicted the change in the opioid admission rate. Oxycodone was the only opioid demonstrating a statistically significant association with the changes in prescription opioid admission rates (beta = 17.58, p < 0.001), with every 100,000 gram increase in oxycodone shipments being responsible for a 17.58% increase in the opioid admission rate change. Moreover, the cross sectional analysis demonstrated that the odds a patient entering an inpatient drug rehabilitation program in a PDMP state was abusing prescription opioids was significantly lower than a patient in a control state (Odds Ratio = 0.775, 95% Confidence Interval 0.764–0.785).

## Discussion

Prescription opioid diversion and abuse, and the adequate treatment of pain are two conflicting public health problems. PDMPs are intended to prevent diversion and abuse while still allowing health care professionals to legitimately and properly manage their patients’ pain. The ARCOS database of prescription opioid shipments per capita, and the TEDS database of prescription opioid admission rates offer the potential to examine the associations between prescription opioid usage and prescription opioid abuse along with the impact of PDMPs on physician prescribing practices, prescription opioid usage, and prescription opioid abuse. Since this study uses analytic ecological and cross sectional designs, the results and conclusions can not, by definition, show definitive associations or causations. However, they can give useful indications of the impact of PDMPs and they can be used to follow future trends.

Our results demonstrate a strong positive association between oxycodone shipments and the prescription opioid admission rate. None of the other three opioids demonstrated statistically significant associations (Tables 2 and 3). Thus, this analysis supports the commonly held belief that increasing oxycodone medical usage contributes to increasing prescription opioid diversion and abuse of prescription opioids. This result also validates the usefulness of comparing the PDMP and control groups with respect to state prescription opioid shipments with particular attention focused on oxycodone shipments. The PDMP group demonstrated a clear trend of decreasing oxycodone shipments and decreasing prescription opioid abuse admissions in comparison to the control group (Fig. 4) which we believe was secondary to PDMPs successfully decreasing prescription opioid diversion. The dramatic increase in oxycodone, hydrocodone, and morphine shipments in both the PDMP and control groups during the study period argues against a PDMP “chilling effect” (Fig. 4). The eleven confounding variables evaluated showed no statistically significant effect on prescription opioid abuse.

For purpose of this paper, we restricted the period studied from 1997 to 2003 due to the following reasons. First, the PDMP group of states did not change throughout the 7 year span with the exception of Kentucky which was instituted in 1998. From 2004 to 2008, 24 additional states instituted a functional PDMP.^4^ We believe that a statistical analysis including these additional years will be much weaker since the PDMP group will be changing too frequently with many of the states’ program durations being less than two to three years.

We also limited our analysis to only four opioids: oxycodone and morphine (Schedule II) and hydrocodone and codeine (Schedule III). Oxycodone and hydrocodone are the most highly used and abused prescription opioids in the schedule II and schedule III classes respectively and are good indicators of PDMPs impact on the flow of prescription opioids into states.^7^,^17^,^18^ Morphine and codeine are the next most commonly used schedule II and III prescription opioids respectively so their evaluation provides a validation of the oxycodone and hydrocodone analysis.

### Limitations of the conceptual model

There are potential weaknesses in our conceptual model that have to be considered while interpreting the results of this paper:

1. We assume that the ARCOS and TEDS databases represent prescription opioid usage and prescription opioid abuse respectively. The limitations of these databases are discussed below.
2. We identified the potential group of confounding variables that we consider important in this model. There may be other confounding variables which we have failed to identify.
3. We make the assumption that since there was a dramatic increase in opioid shipments in both the PDMP and non-PDMP group during the investigation period, this indicates that there was no real chilling effect in the PDMP states. With this assumption in mind, we also assume that the decrease we do see in oxycodone shipments in the PDMP states is secondary to decreased diversion.
4. Burglary has been described as a real source of diversion.^2^

However, in our model, it is not possible to distinguish between diversion of opioids secondary to burglary from pharmacies and diversion of opioids secondary to misused physician prescriptions. We assume that there will be some uniformity between these two types of diversions in PDMP and non-PDMP states. This assumption may not be accurate.

### Limitations of ARCOS data

The ARCOS data have clear limitations. First, the yearly statistics include shipments to hospitals and vetinarians. We made the assumption that vetinarians do not commonly utilize the prescription opioids evaluated in this study and that hospital usage will remain reasonably stable or at the very least, will make a small contribution to any increasing year over year prescription opioid shipments as demonstrated by statistics in the ARCOS database which document national shipments of prescription opioid shipments to hospitals. Secondly, ARCOS does not include prescription opioids which enter states undetected by the surveillance system. For example, illegal internet purchase of opioids is a significant source of prescription opioids and access to prescription opioids across U.S. borders are unaccounted sources.^19^,^20^ As mentioned above, the ARCOS database should ideally report four datasets: i) state shipments minus retail mail order pharmacy shipments, ii) state retail pharmacy shipments, and iii) hospital shipments and iv) veterinary retail pharmacy shipments. This would create a comprehensive and more useful dataset for the evaluation of medical opioid usage and diversion.

### Limitations of TEDS data

Limitations of the TEDS database relate to how it reflects overall prescription opioid abuse. There are a number of parameters which can affect treatment admissions which confound the relationships between diversion, abuse, and admission statistics. These include the willingness of the person to enter treatment, the availability of treatment units in each state, and the insurance coverage for admission to an inpatient treatment unit. The National Survey on Drug Use and Health (NSDUH) documented 11,671,000 persons using prescription opioids for non-medical use while the total prescription opioid admissions in 2003 equaled 50,946.^21^,^22^ The TEDS database only includes the inpatient admissions for drug abuse and thus does not represent the complete population of opioid abusers. Also, it does not distinguish multiple admissions of the same person in the same year. However, since most health insurance coverage for inpatient prescription opioid abuse would not cover a second admission in the same year, this is likely to be a limited issue. Also, the TEDS data only includes admissions that occurred in the publicly funded substance abuse treatment facilities and thus does not represent the entire pool of substance abuse admissions. The public funding constraints may also direct the states to selectively target special populations like pregnant women and adolescents. These limitations of the TEDS data have to be considered while interpreting the results. (Office of Applied Studies and Substance Abuse and Mental Health Services Administration) If the NSDUH published state level data on outpatient management of prescription opioid abuse, this would compliment the TEDS database and improve assessing trends in opioid abuse.

The PDMP states had lower oxycodone shipments per capita, a lower percent increase in their prescription opioid admission rates and lower odds that patients entering treatment programs are abusing prescription opioids, which suggests that PDMPs successfully decrease prescription opioid diversion and abuse. Furthermore, the significant increase in PDMP oxycodone, hydrocodone and morphine shipments from 1997 to 2003 dispels the notion that PDMPs have a significant chilling effect on physician prescribing.

In summary, we have discussed the inherent limitations of both the ecological analytic design and the public surveillance systems utilized. With that said, the results of our study demonstrate a logical progression. That is, increasing prescription oxycodone shipments have a statistically significant strong positive association with increasing prescription opioid abuse admissions and the PDMP group which demonstrates a strong decreasing trend in prescription opioid shipments compared to the control group also demonstrates a decreasing trend in prescription opioid admissions as compared to the control group. These findings support our conclusion that PDMPs decrease diversion of prescription opioids.

## Conclusion

As of November 2008, thirty-two states in the U.S. have operational PDMPs and the National All Schedules Prescription Electronic Reporting Act of 2005 (NASPER) has not yet been funded.^12^ The NASPER legislation, if implemented in full form, would not only establish PDMPs in all 50 states but would also allow health professionals to access PDMP databases in neighboring states allowing for the identification of doctor shoppers crossing state lines to fraudulently obtain controlled substance prescriptions for the purposes of diversion. The fact that some states have as many as one in ten of their inpatient drug rehabilitation patients abusing prescription opioids and the fact that the NSDUH shows more and more teenagers abusing prescription opioids^23^ illustrate the importance of implementing programs which decrease prescription opioid diversion and abuse. Although it is imperative that physicians continue to legitimately prescribe opioids to treat chronic pain, it is important to acknowledge the rise in prescription opioid abuse. Surveillance systems such as PDMPs can successfully deter prescription opioid diversion and abuse. This study supports the efficacy of PDMPs and provides statistical support for establishing PDMPs in all states. The results of this study should also allay the fears of health professionals that PDMPs will exert an undue chilling effect on physician prescribing practices.

## Figures and Tables

**Table 1:** Comparison of the potentially confounding socio-economic factors in the PDMP and control states.

Confounding factor	PDMP 14	Control group	p value
Mean population in year 2000	4,363,473	88,403,550	0.11
Median age in years	35.93	34.50	0.01
Males per 100 females	93.89	94.693	0.51
White (%)	77.99	70.136	0.09
Black/African American (%)	10.79	7.60	0.16
Hispanic/Latino (%)	6.24	11.75	0.05
Female head of household families (%)	11.35	11.82	0.41
Population with atleast HS education (%)	82.32	80.98	0.36
Median household income (U.S. $)	40765.47	42928.57	0.28
Families below poverty level (%)	11.89	12.07	0.86
Population (≥16 yrs of age) unemployed (%)	5.44	5.86	0.21
Population density/Square mile	251.72	155.51	<0.01
Housing unit density/Square mile	102.74	65.18	<0.01

**Abbreviations:** PDMP, prescription drug monitoring program; HS, high school.

**Table 2:** Multiple variable regressions of opioid shipments versus changes in prescription opioid admission rate since 1997.

Model parameters	Beta	p value	95% CI	
Oxycodone shipments	17.58	<0.001	13.99	21.18
Hydrocodone shipments	0.02	0.987	−2.99	3.05
Codeine shipments	−6.17	0.006	−10.51	−1.83
Morphine shipments	−10.07	0.063	−20.69	0.55

**Abbreviation:** CI, confidence interval.

**Table 3:** Multiple variable regressions of oxycodone and codeine shipments and the potential confounding factors versus changes in prescription opioid admission rate since 1997.

Model parameters	Beta	p value	95% CI	
Year	17.808	<0.001	10.399	25.218
Oxycodone shipments	9.689	<0.001	6.018	13.359
Codeine shipments	−4.196	0.073	−8.778	0.387
Median age	−3.091	0.395	−10.241	4.060
Percentage of population aged 25–44 years	5.634	0.407	−7.731	18.998
White	0.713	0.415	−1.008	2.434
Black/African American	−1.035	0.446	−3.703	1.634
Hispanic/Latino	−1.347	0.123	−3.061	0.367
Male: Female ratio	−5.274	0.066	−10.908	0.360
Percentage of female head of household families	9.627	0.421	−13.909	33.162
Median household income	0.001	0.776	−0.006	0.008
Percentage of population below poverty level	6.637	0.329	−6.736	20.010
Percentage of population (≥16 yrs of age) unemployed	−1.256	0.805	−11.292	8.781
Percentage of population with at least HS education	3.743	0.174	−1.660	9.145
Population density/Square mile	−0.082	0.876	−1.124	0.959
Housing unit density/Square mile	0.104	0.936	−2.452	2.661

**Abbreviations:** CI, confidence interval; HS, high school; PDMP, prescription drug monitoring program.
